# Integration of ‘omics’ data in aging research: from biomarkers to systems biology

**DOI:** 10.1111/acel.12386

**Published:** 2015-08-30

**Authors:** Jonas Zierer, Cristina Menni, Gabi Kastenmüller, Tim D. Spector

**Affiliations:** ^1^ Department of Twins Research and Genetic EpidemiologyKings College LondonLondonUnited Kingdom; ^2^ Institute of Bioinformatics and Systems BiologyHelmholtz Zentrum MünchenNeuherbergGermany

**Keywords:** data integration, graphical models, high‐throughput data, omics, systems biology

## Abstract

Age is the strongest risk factor for many diseases including neurodegenerative disorders, coronary heart disease, type 2 diabetes and cancer. Due to increasing life expectancy and low birth rates, the incidence of age‐related diseases is increasing in industrialized countries. Therefore, understanding the relationship between diseases and aging and facilitating healthy aging are major goals in medical research. In the last decades, the dimension of biological data has drastically increased with high‐throughput technologies now measuring thousands of (epi) genetic, expression and metabolic variables. The most common and so far successful approach to the analysis of these data is the so‐called reductionist approach. It consists of separately testing each variable for association with the phenotype of interest such as age or age‐related disease. However, a large portion of the observed phenotypic variance remains unexplained and a comprehensive understanding of most complex phenotypes is lacking. Systems biology aims to integrate data from different experiments to gain an understanding of the system as a whole rather than focusing on individual factors. It thus allows deeper insights into the mechanisms of complex traits, which are caused by the joint influence of several, interacting changes in the biological system. In this review, we look at the current progress of applying omics technologies to identify biomarkers of aging. We then survey existing systems biology approaches that allow for an integration of different types of data and highlight the need for further developments in this area to improve epidemiologic investigations.

## Introduction

Aging is often described as the progressive accumulation of changes with time leading to a loss of physiological aptitude and fertility, an increased susceptibility to disease and ultimately to death (Harman, 1988, 2001; Kirkwood & Austad, 2000; Vijg & Suh, 2005; López‐Otín *et al*., 2013). Despite considerable effort and the development of many theories, the underlying process is still largely unknown (Kirkwood & Austad, 2000; Weinert & Timiras, 2003; Rattan, 2006).

Researchers distinguish between chronological and biological age. Chronological age is defined as the absolute time that an individual lives. In contrast, biological age is a broader concept that takes the individual physical and mental health into account, thus capturing individual differences of the aging process. Most aging studies search for associations of chronological age with clinical and molecular phenotypes (Warming *et al*., 2002). However, several studies used phenotypes, such as lung function, grip strength or bone mineral density, as proxies to investigate molecular changes in biological aging (Jackson *et al*., 2003; Bell *et al*., 2012; Levine, 2013). Researchers also investigated reasons of retarded biological aging and longevity by comparing centenarians with younger controls (Biagi *et al*., 2012; Sebastiani *et al*., 2012).

The life expectancy in the UK increased by 5.3 years for men and 4.7 for women over the last two decades and is predicted to further increase in the next twenty years (Oeppen & Vaupel, 2002; Office for National Statistics 2014). With increasing life expectancy, age‐related diseases are expected to rise dramatically (700 000 people suffered from dementia in 2000, 800 000 in 2012 and approximately 1 million people will be affected by dementia in 2021 (Alzheimer's Society 2014)) with major impacts on healthcare costs. Thus, a better understanding of aging and its influence on disease is a long term public health goal and a hot topic of current medical research.

Omics technologies provide valuable tools to study aging on the molecular level. Reductionist data analyses, testing the measured variables separately for association with age, have been extensively applied. Such studies successfully identified hundreds of epigenetic mutations, gene expression levels, metabolite concentrations to be linked with chronological and/or biological age (see below for details). Even though these results improved our understanding of aging as a complex phenotype, the mechanisms underlying these associations and the impact of interactions between different biological entities remain elusive in most cases. In contrast to reductionist approaches, systems biology aims to analyse all components of a biological process simultaneously taking into account their interactions and their intrinsic hierarchical structure (Ideker *et al*., 2001; Barabási & Oltvai, 2004). With more and more high‐throughput data becoming available, systems biology has led to many new methods and their successful application on age and age‐related phenotypes (as outlined below).

In this review, we will briefly summarize the current progress in ‘omics’ technologies and their application in aging research. We will then highlight some problems of the reductionist approach and discuss how these may be overcome using systems biology. We present a selection of statistical methods used in systems biology along with their current and possible future applications in the field of aging research to move from biomarkers of aging to a more holistic understanding of the aging process.

## Omics and aging

New technologies allow the measurement of ‘omics’ data and numerous association studies have been conducted. Valdes *et al*. (2013) thoroughly reviewed the application of these technologies to identify molecular markers of aging from each omics level. Therefore, the following section will only briefly highlight some key results and concentrate on recent findings.

### Genomics

Genomics was the first omics field for which high‐throughput measurements became available. Current chips are able to measure up to 5 million single nucleotide polymorphisms (SNPs) (Ha *et al*., 2014). Today, next‐generation sequencing technology is slowly replacing the chip technology as the cost of sequencing has dropped below $0.10 per million bp (Liu *et al*., 2012). Thus, gene variation is nowadays often available at single nucleotide resolution.

While aging (or rather longevity) itself was found to be only about 20% heritable (Murabito *et al*., 2012), many age‐related diseases are highly heritable. For instance, Alzheimer's disease (AD) shows a heritability above 70% (Gatz *et al*., 2006) and osteoarthritis (Ishimori *et al*., 2010) or cataract show 50% heritability (Hammond *et al*., 2001).

The GenAge database contains about 300 human candidate genes for aging based on homology with model organisms (Tacutu *et al*., 2013). Sebastiani *et al*. (2012) recently published a refined model consisting of 281 SNPs to distinguish between centenarians and younger controls in a cohort of 1715 people. One of these SNPs is located in ApoE, which is so far the only gene that has been reliably associated with longevity at genomewide significance level (Deelen *et al*., 2011; Nebel *et al*., 2011). Common genetic variants at this locus have been associated with accelerated aging and cognitive decline (Johnson, 2006; Davies *et al*., 2014), possibly by increasing the risk for coronary artery disease, stroke and AD (Smith, 2002). Even though some studies provided evidence that mutations of FOXO transcription factors are related to longevity (Willcox *et al*., 2008; Flachsbart *et al*., 2009), as well, GWASs failed to replicate this at the level of genomewide significance.

### Epigenomics

Epigenomics describes the study of heritable changes in the genome that are not caused by DNA sequence mutations (Lodish, 2013). The most common epigenetic mechanism is DNA methylation, which is known to often silence gene expression. In contrast to the genome, which is the same in all cells, the epigenome is an important factor of cell differentiation leading to profound epigenetic differences across different cell types (Meissner, 2010). The current methylation chip by Illumina measures over 485 000 methylation sites and covers 99% of all RefSeq genes (Illumnia 2011). However, it covers less than 10% of variable regions (Ziller *et al*., 2013).

The epigenome is influenced by environmental and lifestyle factors (Nakajima *et al*., 2010; Alegría‐Torres *et al*., 2011; Breitling *et al*., 2011) and is associated with many complex diseases such as neurodegenerative disorders (reviewed by Portela & Esteller, 2010) and cancer (Ehrlich, 2002; Horvath, 2013). Nearly 500 differentially methylated regions were found to be associated with chronological age and age‐related phenotypes such as lung function, cholesterol levels and maternal longevity (Bell *et al*., 2012). A recent study by Weidner *et al*. (2014) showed that methylation patterns of just three sites are sufficient to predict chronological age. Thus, many of the previously identified methylation sites might not be independently associated with age. Interestingly, variation in methylation with age is consistent across several tissues and cell types (Horvath, 2013). Together, they form a global pattern of hypomethylation in repetitive sequences, hypermethylation in promoter regions and higher intercell variability (Cevenini *et al*., 2008; Bacalini *et al*., 2014). Besides DNA methylation, other epigenetic changes, such as histone methylation and acetylation, have been found to be associated with longevity in model organisms (Dang *et al*., 2009; Greer *et al*., 2010). Investigating these modifications in humans could shed light on so far unknown mechanisms of aging.

### Transcriptomics

Genes are transcribed into RNA molecules, which are further processed in a tightly controlled process. The entirety of the RNA transcripts is referred to as transcriptome. It can be divided in coding RNAs, which are further translated in proteins, and noncoding RNAs, which perform various functions, such as regulation of gene expression (Eddy, 2001). Transcript abundances can be measured by either chips or sequencing methods.

Similar to the epigenome, gene expression was shown to dramatically change with age. A pioneer study comparing postmortem human frontal cortex tissue samples between 30 individuals of different ages yielded 463 differentially expressed genes (Lu *et al*., 2004). Despite the small sample size, results were replicated in subsequent experiments. Four years later, Berchtold *et al*. (2008) identified several thousand age‐related changes in gene expression in four different brain tissues. Later studies by different groups identified profound changes in the transcriptome with age in further tissues, such as skin, adipose tissue (*N* = 865) (Glass *et al*., 2013) and kidney (*N* = 134) (Rodwell *et al*., 2004). Most of these changes did not overlap in different tissues. A meta‐analysis across different species and tissues revealed only 73 genes consistently associated with age (de Magalhães *et al*., 2009). This suggests that most observed age‐related changes in the transcriptome are either species and tissue specific or false‐positive discoveries (reviewed by Valdes *et al*., 2013). In their meta‐analysis, genes related to immune response and lysosome tended to be overexpressed, while genes related to mitochondria and oxidative phosphorylation were underexpressed in elderly (de Magalhães *et al*., 2009).

### Proteomics

Proteins are translated from coding transcripts. Due to alternative splicing and post‐translational protein modifications, the number of proteins is estimated to be two orders of magnitudes higher than the number of genes (Ginsburg & Haga, 2006). However, current proteomic techniques based on immunoassays, protein arrays or mass spectrometry can measure only a small fraction of the proteome (up to 1000 proteins in a sample). The most comprehensive description of the human proteome across various tissues to date consists of 18 097 proteins (19 376 isoforms) collected from ten thousand mass spectrometry experiments (Wilhelm *et al*., 2014).

Due to these technicalities, ‘proteomics’ studies in aging research so far focused on smaller sets of proteins and small sample sizes. In an early study of protein abundance in the vastus lateralis muscle, Gelfi *et al*. (2006) observed higher abundance of several proteins involved in aerobic metabolism and a lower abundance of proteins involved in anaerobic metabolism in the elderly. Besides this, six transport proteins were consistently underexpressed in older individuals. However, only 12 samples were analysed in this study without replication. A recent study by our group analysed over 1000 proteins in 200 plasma samples using the SOMAscan assay (Menni *et al*., 2015). Eleven proteins were found to strongly associate with chronological age as well as age‐related phenotypes such as lung function and blood pressure. The results were replicated in an independent cohort.

Even though comprehensive proteomics studies are still missing, proteins are likely to be associated with several age‐related diseases. For instance, cardiovascular disease (Mehra *et al*., 2005) and AD (Swardfager *et al*., 2010) are consistently associated with elevated levels of pro‐inflammatory cytokines.

### Post‐translational modifications – glycomics

Post‐translational modifications are important elements of proteins, which can alter their biochemical properties such as protein structure, binding preferences and enzyme activity. There are many different modifications ranging from addition of small molecules (e.g. acetylation or phosphorylation), over addition of larger molecules such as lipids or sugar chains (e.g. palmitoylation, glycosylation), to the addition of whole proteins (e.g. ubiquitination).

The most common modification is glycosylation, which attaches sugar chains to proteins. The attached oligosaccharides – glycans – are supposed to mainly serve as structural elements of proteins or specific binding sites for other glycans or proteins (Varki *et al*., 2009). However, glycans are highly diverse and many of them are not yet characterized or annotated. Thus, glycans might have many additional functions. For example, glycans in the gut act as food for microbes (Koropatkin *et al*., 2012), which could be implicated in immune functions that are important in aging. Recent development allows the high‐throughput measurement of glycans of either a single protein or all proteins simultaneously (Royle *et al*., 2008; Pucić *et al*., 2011).

The application of this technology on epidemiological cohorts revealed that glycan structures are stable for one individual over time (Gornik *et al*., 2009) but very diverse within a population (Knezević *et al*., 2009; Pucić *et al*., 2011). Differences in glycomes were found to be related with various cancers (Fuster & Esko, 2005; Adamczyk *et al*., 2012). Recently, Kristic *et al*. (2013) showed that IgG glycans are strongly associated with age: a linear combination of three glycans explained 58% of the observed variance of chronological age (Kristic *et al*., 2013) in a study of four independent populations with 5117 participants in total.

### Metabolomics

Metabolomics investigates the low‐molecular‐weight molecules in a biological system. The measured molecules are often referred to as metabolites as many of them act as educts, products and intermediates of the cellular metabolism. Currently, the Human Metabolome Database (Wishart *et al*., 2013) contains more than 40 000 distinct metabolites from different tissues. Similar to proteomics, to date, there is no analytical method available to determine and quantify all metabolites in a single experiment. Current platforms, using either chromatography coupled with mass spectrometry or nuclear magnetic resonance, can measure roughly a thousand metabolites in untargeted settings and a smaller number using predefined targeted approaches. The restriction of the targeted approach comes with the advantages of higher sensitivity, absolute instead of relative quantification and straight‐forward compound identification (Patti *et al*., 2012; Tzoulaki *et al*., 2014).

In 2008, the first metabolome‐wide association study on age analysed the plasma metabolome of 269 individuals using an untargeted approach. The authors found 100 of 300 compounds to correlate with chronological age (Lawton *et al*., 2008). More recently, larger cohorts were employed to study the association of metabolites and age using both targeted and untargeted metabolomics platforms. Yu *et al*. (2012) analysed 131 targeted metabolites in 2162 individuals from the KORA study, while we analysed 280 untargeted metabolites in 6055 twins from the TwinsUK cohort (Menni *et al*., 2013b). Both studies identified half of the analysed metabolites to be associated with chronological age. Many of the those metabolites were also found to significantly correlate with age‐related phenotypes such as lung function, bone mineral density and cholesterol levels (Menni *et al*., 2013b), AD (N = 93) (Orešič *et al*., 2011), cancer (reviewed by Teicher *et al*., 2012) and type 2 diabetes (N = 100) (Suhre *et al*., 2010; Menni *et al*., 2013a). One of those metabolites is C‐glycosyltryptophan, a potential degradation product of glycosylated proteins.

### Microbiomics

The human microbiome describes the complete set of microbial species (and their genomes) hosted by the human body. The largest microbial community resides in the gut, where microbial cells and their genes outnumber human cells (10:1) and genes (100:1) (Peterson *et al*., 2009; Zhu *et al*., 2010; The Human Microbiome Project 2014a). More than 10 000 different species with millions of protein‐coding genes were identified by the Human Microbiome Project (Turnbaugh *et al*., 2007; Peterson *et al*., 2009; Biagi *et al*., 2012) and >1000 of these microbes have so far been fully sequenced (The Human Microbiome Project 2014b). Although twin studies have found a modest genetic influence on some phyla, most of the variation is environmental (Goodrich *et al*., 2014).

The composition of the microbe flora varies a lot across individuals (Turnbaugh *et al*., 2007; Zhu *et al*., 2010) and even between different parts of the body (Kong, 2011). It has a huge influence on many biological processes such as immune response, metabolism and disease (Zhu *et al*., 2010; Grice & Segre, 2012). While the microbiome seems to be relatively stable during adulthood, it changes significantly in later life (Guigoz *et al*., 2008; Biagi *et al*., 2010; Claesson *et al*., 2011). Biagi *et al*. (2010) observed drastic changes in the gut microbiome of centenarians compared with young adults as well as elderly, namely a general loss of diversity and increased abundance of bacilli and proteobacteria. The latter were reported to promote inflammation under certain conditions (Round & Mazmanian, 2009). Similar findings were revealed in other elderly populations, which also considered the dietary and residential situation of elderly patients (Claesson *et al*., 2012).

### Phenomics

Simultaneously with omics data, the dimension of clinical and lifestyle traits, particularly clinically used intermediate traits, keeps increasing. Epidemiological studies collected thousands of clinically relevant phenotypes beyond omics data types. These range from anthropometric measures to health and lifestyle questionnaires (Moayyeri *et al*., 2013). Collecting high‐dimensional clinical data is important to unveil pleiotropy of genes and interactions amongst clinical phenotypes such as comorbidities (Houle *et al*., 2010). Driven by omics technologies, statistical and bioinformatic methods to analyse high‐dimensional data are becoming available. These facilitate the investigation of numerous clinical phenotypes in parallel, thus defining the new field of *phenomics* (Houle *et al*., 2010).

Phenomics is especially important for aging research. Dozens of clinical phenotypes, such as Parkinson's (Reeve *et al*., 2014), AD (McAuley *et al*., 2009), body mass index, blood pressure (Mungreiphy *et al*., 2011) and bone mineral density (Warming *et al*., 2002), as well as lifestyle parameters, such as nutrition (Wieser *et al*., 2011), smoking and physical activity, are strongly related to age (Harman, 1988; Wang *et al*., 2009). Composite measures such as the Rockwood frailty index (Rockwood & Mitnitski, 2007) combine several of those clinical traits to form a more homogenous phenotype – frailty – from its diverse appearance. Such frailty measures can be considered as measures for biological age (Mitnitski *et al*., 2013). Many of these (and other) clinical phenotypes correlate or even depend on each other (McAuley *et al*., 2009; Baylis *et al*., 2014). Only extensive collection of data and their joint analysis will help to unveil these dependencies and find causal relationships.

## From omics to systems biology

**Figure 1:**
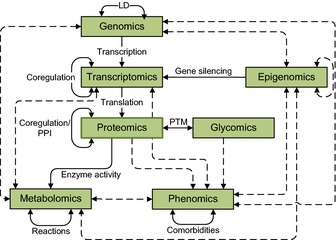
Interdependencies of omics data: The figure illustrates dependencies which can be observed within almost any omics data set. Solid lines indicate biological processes which cause dependencies, while dashed lines represent observed associations.

Most of the studies summarized above concentrated on the bivariate associations of age (or age‐related diseases) with one type of omics data. However, there are strong interdependencies within and between the different omics data (see Fig. 1).

Correlations can be observed practically between all levels of biological organization. Following the central dogma of molecular biology, genomics, transcriptomics and proteomics are correlated ‘by definition’. Furthermore, metabolite concentrations are influenced by genetic variants (Shin *et al*., 2014) and epigenetic factors (Petersen *et al*., 2014) mediated through changes in gene expression or enzyme activity. Methylation levels do not only influence the gene expression (Jaenisch & Bird, 2003), but are also correlated with gene variants (Bell *et al*., 2012) and environmental factors (Breitling *et al*., 2011). Our group has recently demonstrated that even the microbe composition is partly under host genetic influence (Goodrich *et al*., 2014). Similarly, all levels of omics data are influenced by genetics as well as by environment and aging. Correlations, however, do not only occur between but also within each type of data. For instance, in genomics linkage disequilibrium, the correlated occurrence of SNPs is a ubiquitous phenomenon. Transcription factors often coregulate the expression of multiple genes (Allocco *et al*., 2004), and methylation patterns of neighbouring CpG sites were reported to be correlated (Bell *et al*., 2012). Metabolites are linked by a network of biochemical reactions, causing strong correlations between them (Krumsiek *et al*., 2011). Even phenotypes often cluster. Comorbidities, the over proportional co‐occurrence of diseases, were shown to affect many diseases possibly through shared underlying mechanisms (Goh *et al*., 2007).

These biological correlations can confound the associations and this is a major issue of current research. For instance, 153 metabolites were found by our group to be associated with age, but subsequent analyses showed that only 22 of them are associated with age independently (Menni *et al*., 2013b). Similarly, 21 of 24 measured IgG glycans were correlated with age, but only 3 of them explain 58% of the variance (Kristic *et al*., 2013). The same was found for epigenetic data (Weidner *et al*., 2014). Huge lists of associations with aging are being unveiled using all kinds of data, but the biologically interesting, causal associations are often obscured by this wealth of results. Approaches taking simultaneously information from all omics levels into account are needed to reconstruct the processes involved in aging on a systems level (Valdes *et al*., 2013).

Even though high‐throughput technologies are advancing and more and more data are becoming available, integration of omics remains a challenging problem. Besides the restricted availability of multi‐omics data sets for the same samples, technical limitations hamper the integration process. While genomics and transcriptomics are able to measure the entire set of variants, other omics (e.g. proteomics and metabolomics) measure only a small fraction of all entities. Many high‐throughput technologies suffer from considerable technical variation and strong batch effects. Stringent quality control and thorough data normalization are crucial when analysing this type of data. Furthermore, the complexity of the organism has to be taken into account. While the genome is more or less stable, all other levels of omics change between cell types and over time. Many samples, such as whole blood, contain a mixture of different cell types with potentially different epigenomes, transcriptomes (Houseman *et al*., 2012; Jaffe & Irizarry, 2014). Finally, different organs and cells influence each other. The blood metabolome, for instance, is heavily influenced by processes occurring in the liver or in other organs, and multitissue samples are needed to fully understand these. This in turn is not always feasible in an epidemiological setting as collection of tissues often involves invasive procedures. Nevertheless, data integration is an important and active field of research. A first step of data integration is the integration and joint interpretation of separate results. The Digital Ageing Atlas (Craig *et al*., 2014) summarizes more than 4000 age‐related changes across different technologies to facilitate systems‐level analyses of aging.

### Introduction to systems biology

**Figure 2:**
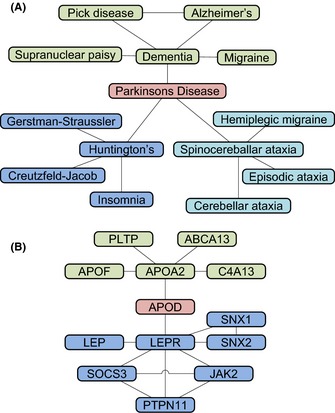
Topological Properties of Biological Networks (A) is an excerpt from the human disease network (Goh *et al*., 2007). Nodes represent diseases; these are connected if they are associated with the same gene. Parkinson's disease connects three isolated disease clusters (colours), thus having a low clustering coefficient (0%) and high betweenness (72%). (B) is the close neighbourhood of the ApoD protein in a PPI network from STRING DB (Franceschini *et al*., 2013) using only experimentally confirmed interactions. ApoD connects two clusters and is, despite the low degree (2) and clustering coefficient (0%), a central node (betweenness centrality: 53%). In contrast, LEPR is central within the blue cluster (degree: 7, clustering: 14%).

The aim of systems biology is to understand the system and its functions as a whole rather than as separate components (Cassman, 2005), with the final objective to mathematically model biological systems and simulate their outcomes. As a first step, the complex interactions and dependencies between these components must be formally described to enable systematic analysis and simulation of the biological system of interest. A technique widely used in systems biology is to translate biological interactions into mathematically well‐defined networks (graphs). For instance, metabolites interact in chemical reactions, thus forming a network in which *nodes* describe the metabolic compounds and *edges* indicate chemical reactions. Similarly, transcription factors bind DNA to control gene expression, forming the gene regulatory network (GRN) and interacting proteins build a protein–protein interaction network (PPI) (cf. Fig. 2B). These networks interact, making data integration an important aspect of systems biology. One example for a phenotypic network was created by Goh *et al*. (2007) using diseases as nodes and connecting diseases with shared genetic risk factor by edges (cf. Fig. 2A). By doing so, they showed that many disorders share a set of underlying genetic risk variants and that similar diseases are caused by similar genes.

Graphs can be explored using a variety of established algorithms. One common task is the identification of modules, that is subgraphs in which nodes share certain properties. In biological networks, modules correspond to functional units, such as the glycolysis pathway in the metabolic network. The modules are usually interconnected and together form a hierarchical structure in which the distribution of node degrees – the number of edges per node – follows the power law (Barabási & Oltvai, 2004). Hence, most nodes have only few connections and few nodes have many connections. These highly connected nodes are called hubs (Albert *et al*., 2000; Jeong *et al*., 2001). Several other measures exist to describe the topology of networks and topological features of nodes. For example, the clustering coefficient measures how densely the neighbourhood of a node is connected and thus highlights nodes which are central within a cluster (e.g. LEPR in Fig. 2A). Another measure is the betweenness centrality, which measures the proportion of pairwise shortest paths containing a node. It thus quantifies the importance of a node for connecting other nodes from different modules (e.g. Parkinson's disease in Fig. 2A and APOD in Fig. 2B). The highly connected, central nodes are thought to be key players in the system, connecting several modules and controlling network fluxes. They were shown to be of particular importance for many diseases and survival of the organism (Barabási & Oltvai, 2004; Joy *et al*., 2005; Yu *et al*., 2007).

Many software packages for graph analysis and visualization are publicly available. For instance, the R package igraph (Csardi & Nepusz, 2006) or the standalone program Cytoscape (Shannon *et al*., 2003) can be used to analyse and visualize graphs. Cytoscape also provides easy integration of biological databases such as Gene Ontology (Ashburner *et al*., 2000), Reactome (Croft *et al*., 2014), the Kyoto Encyclopaedia of Genes and Genomes (KEGG) (Kanehisa & Goto, 2000) or BioGRID (Chatr‐Aryamontri *et al*., 2013) by third‐party apps. Several methods were developed to identify modules of nodes which are jointly affected by the condition of interest. Two publicly available examples are the Cytoscape plugin jActiveModules (Ideker *et al*., 2002) and the R package BioNet (Beisser *et al*., 2010).

Here, we present a selection of current methods to construct and analyse biological networks as an approach to systems biology and their impact on aging research.

### Enrichment and network topology analysis in predefined networks

A popular approach to put the results of an association study in a systems biology context is projecting the variables of interest – such as age‐related genes, proteins or metabolites – onto known biological (reference) networks. The neighbourhood of these target variables and their topological properties can then be assessed using the experimentally predefined PPI, GRN or metabolic networks. Instead of interpreting individual entities separately, *a priori* knowledge about their interactions and common functions can be used to identify modules that are jointly affected by the condition of interest.

Several databases offer a collection of experimentally identified interactions that can be used as predefined reference networks for enrichment and topology. In case of PPI, the Human Protein Reference Database provides more than 40 000 PPIs (Keshava Prasad *et al*., 2009), the Database of Interacting Proteins more than 7000 interactions (Xenarios *et al*., 2002) and the MIPS mammalian protein–protein database roughly 1000 hand‐curated interactions of human proteins (Pagel *et al*., 2005). GRN are provided by the ChIPBase (Yang *et al*., 2013), which contains six million transcription factor binding sites from >300 experiments. Metabolic reactions are amongst others provided by KEGG.

Enrichment analysis is a convenient way to incorporate existing knowledge from biological reference networks without analysing graph topology directly. Therefore, predefined (functional) modules within the reference networks are used to test overrepresentation of associated genes, proteins or metabolites in these groups. When investigating genes, researchers usually use Gene Ontology to group genes based on *biological processes*,* molecular functions* or *subcellular localization*. For metabolites, the KEGG and Reactome databases provide curated information about biochemical pathways. The R packages GSEABase, GAGE (Luo *et al*., 2009) and the webservice MSEA (Xia & Wishart, 2010) are just some of many available implementations and variations in the original gene set enrichment analysis (Subramanian *et al*., 2005) algorithm.

In aging research, enrichment analysis unveiled an overexpression of genes involved in immune response, lysosome and glycoproteins and an underexpression of mitochondrial‐ and oxidative phosphorylation‐related genes in old people compared with young (de Magalhães *et al*., 2009). In human brain tissue, oxidative stress/DNA repair and inflammation‐related genes were shown to be enriched in the set of differentially expressed genes between young and old individuals (Lu *et al*., 2004). Enrichment analysis facilitates the identification of pathways that are important for the aging process. It thus helps to make sense out of the individual associations and find biological interpretations for the observed molecular changes.

To become independent of predefined module annotation and to enable more detailed network analysis, the variables of interest can also be mapped directly on the known PPI, GRN or metabolism networks. Modules can then be identified dynamically based on the measured data. Moreover, additional topological properties of the variables of interest can be assessed.

Studying human PPI networks revealed that genes that are associated with aging by homology have higher node degrees and higher betweenness centrality compared with other genes (Bell *et al*., 2009). Furthermore, aging‐related genes are not spread throughout the interactome, but cluster in few tightly connected modules. These modules were enriched in DNA damage repair and stress response genes (Kriete *et al*., 2011). The high connectivity of aging genes was used by Tacutu *et al*. (2012) to select neighbours of longevity‐related genes in a PPI network as longevity‐gene candidates. Subsequent experiments in *C. elegans* revealed 30 new longevity‐associated genes, proving the potential of network biology for candidate gene selection. Using a modified PPI network, Wang *et al*. (2009) showed a tight connection of the genetic causes of aging and disease. These results indicate that aging does not occur due to random errors but is an organized process. Another PPI‐based approach to data integration was developed by West *et al*. (2013). They incorporated epigenomic data by assigning DNA methylation sites to each protein in the graph and then identifying modules of differentially methylated genes/proteins in the resulting network. By doing so, they avoided predefined gene sets as used by enrichment analysis. The analysis revealed three differentially methylated modules, which were replicated across several tissues. Two of them contained mainly transcription regulating genes, while the third one contained genes related to stem cell differentiation.

A drawback of experimentally derived PPI or GRN is that such methods detect up to 50% false positives while many true interactions are missed (Huang & Bader, 2009; Marbach *et al*., 2012). Even more importantly, those reference networks completely ignore the tempo‐spatial properties of the interactions. This restricts results to already observed, possibly inactive interactions. One method to overcome the static nature of PPI networks are Negative–Positive (NP) networks (Xia *et al*., 2006). These integrate the PPI network with transcriptomics data by restricting it to edges between (anti‐)correlated proteins/genes. Therefore, only those interactions (=edges) that are active under the observed condition are further analysed. Xue *et al*. (2007) applied this method to the previously mentioned data set of brain gene expression and unveiled two anticorrelated modules containing cell proliferation‐ and cell differentiation‐related proteins. Two other modules consisting of protein processing and immunity‐related genes, respectively, were found to be slightly correlated with the cell proliferation module. A recent study went one step further and restricted a PPI network to highly expressed genes in different stages of aging for each sample separately, thus generating a set of *dynamic binding networks* instead of a single network. Even though the global properties of all those graphs were very similar, the centrality of several genes correlated with age (Faisal & Milenković, 2014).

Incorporating biological networks to analyse aging‐related changes showed the tight connection of aging and disease on a molecular level. Furthermore, it has been shown that aging affects central genes, which are important for the network integrity (Bell *et al*., 2009). While network‐based enrichment and analysis using PPI networks is common for genetic and transcriptomics data, it has not been applied to aging studies using metabolomics data. This could be a promising approach to systematically identify metabolic pathways jointly affected by the aging process.

### Analysis of data‐derived networks

Despite their successful applications, all approaches presented so far rely on predefined, static networks. To overcome the limitations of such networks, inferring networks directly from the measured data is the next step.

#### Weighted gene co‐expression network analysis

The weighted gene co‐expression network analysis (WGCNA) (Zhang & Horvath, 2005) infers gene–gene interaction networks directly from transcriptomics data. Miller *et al*. (2008) applied this method to the previously mentioned gene expression data set of 30 human frontal cortex samples at different ages and then compared the results with a network derived from an AD transcriptomics study. It revealed significant overlap between healthy aging and AD, suggesting that there might be a shared molecular basis for both processes. Three AD network modules overlapped with aging network modules, containing mostly synapses‐, transport‐ and transcriptional regulation‐related genes.

#### Gaussian graphical models

Despite the successful application of WGCNA on transcriptomics data, Krumsiek *et al*. (2011) showed that ordinary correlations are not suitable to analyse metabolomics data from large cohort studies. They analysed metabolite concentrations of >1000 samples and found that more than half of all pairs of 151 metabolites correlated significantly, even when using a restrictive Bonferroni correction at an alpha level of 0.01. This is largely due to indirect associations, which cannot be distinguished from direct associations by the Pearson correlation coefficient. Graphical models (GMs), also known as conditional independence graphs, were proposed to overcome this problem and infer biological meaningful networks from metabolomics (Steuer, 2006; Krumsiek *et al*., 2011) as well as other omics data (de la Fuente *et al*., 2004; Yuan *et al*., 2011; Mangin *et al*., 2012). GMs are probabilistic models where an edge between two variables illustrates their *conditional dependence* given all other variables in the model. Implicitly, the absence of an edge represents the conditional independence of the according variables. Several algorithms to infer GMs from purely binary data are publicly available as R packages (Wainwright *et al*., 2006; Höfling & Tibshirani, 2009; Guo *et al*., 2010; Ravikumar *et al*., 2010). Their counterparts for purely continuous data are Gaussian graphical models (GGMs), which use partial correlations to infer graphs. A partial correlation of two variables X and Y conditioned on a set of variables Z quantifies the portion of the correlation between X and Y which cannot be attributed to Z. Several algorithms exist to infer GGMs (d'Aspremont *et al*., 2006; Meinshausen & Bühlmann, 2006; Yuan & Lin, 2007; Friedman *et al*., 2008; Mazumder & Hastie, 2012). Several of them, such as the well‐established graphical lasso (Friedman *et al*., 2008; Mazumder & Hastie, 2012), use regularization to further reduce the number of edges in the graph. This allows researchers to concentrate on fewer high‐confidence interactions.

Gaussian graphical models can reconstruct biological pathways from metabolomics and transcriptomics data, but have not yet been applied in aging research. However, their application could help reduce the ‘overabundance’ of results to fewer, meaningful associations. The major drawback of GMs is that they can only be used for pure Gaussian or pure binary data. Shin *et al*. (2014) overcame this problem by first constructing a GGM from metabolite concentrations and then adding gene variants as nodes and connecting them with associated metabolites. The resulting network illustrates the genetic control of the metabolism in an intuitive way. However, it is no longer a GM, and edges do not indicate conditional independence any more.

#### Mixed graphical models

Recent developments allow the integration of different types of data while maintaining the favourable properties of GGMs, namely mixed graphical models (MGMs) (Tur & Castelo, 2012; Chen *et al*., 2013; Fellinghauer *et al*., 2013; Lee & Hastie, 2015). Fellinghauer *et al*. (2013) proposed a very flexible algorithm based on stability selection (Meinshausen & Bühlmann, 2010). It makes use of established methods such as random forests or regression models to rank interactions between variables of different types. Thus, it can handle many different data types such as disease states, metabolite levels and gene variants. Due to the usage of stability selection, it has an intrinsic error control. MGMs provide a powerful tool for multivariate analyses of high‐dimensional data, but have not been applied in biological research, yet. Their application could shed light on the complex relationship between aging and disease.

Gaussian graphical models as well as MGMs are undirected models. Therefore, neither of them can be used to infer causal direction. In epidemiological research, Mendelian randomization is a common approach to infer causality from observational data. It takes advantage of the invariability of gene variants to separate the study population in groups, thus mimicking a randomized controlled trial (for further details, see Brion *et al*., 2014). Mendelian randomization can be used to further investigate edges of interest that were previously identified by GMs. However, it relies on stable associations with genetic variants and assumes that this genetic variant is not related to any other potential confounding factor. Due to these restrictions, it is not suitable to infer large‐scale networks.

#### Bayesian networks

Another approach that allows inferring causality from observational data under certain assumptions is based on Bayesian networks (BNs). Similar to GGMs, BNs are probabilistic models in which edges represent the conditional independence between variables. However, BNs are DAGs, thus distinguishing between an influence of X on Y and the influence of Y on X. In return, the acyclicity of the causal graph is an assumption which might not hold true for biological networks. The application of BNs on high‐throughput transcriptomics data by Friedman *et al*. (2000) demonstrated the potential of this method to extract biological meaningful associations without prior knowledge. Several different methods are available to estimate the structure of BNs from binary, continuous and even mixed data such as the R packages bnlearn (Scutari, 2010) (Table 1).

**Table 1:** Overview over system biology methods and their application in aging

Method	Prerequisites	Applies to	Availability	Application
Enrichment Analysis	Module definition (e.g. gene sets from Gene Ontology)	Genomics Transcriptomics Proteomics Metabolomics	Several R packages (e.g. GSEABase, GAGE, MSEA), online tools DAVID or Enrichr	Lu *et al*. (2004), de Magalhães *et al*. (2009)
Network Mapping	Predefined network, such as protein–protein interaction (PPI) networks, gene regulatory network (GRN) or metabolic network	Any omics data	R package igraph, Cytoscape with various plugins	Wang *et al*. (2009), Bell *et al*. (2009), West *et al*. (2013), Faisal & Milenković (2014)
NP Networks	PPI Network	Transcriptomics	–	Xue *et al*. (2007)
Weighted Gene Co‐Expression Network Analysis (WGCNA)	–	Transcriptomics (and possibly other continuous data)	R package WGCNA	Miller *et al*. (2008)
Gaussian graphical models (GGMs)	–	Any multivariate Gaussian distributed data	Several R packages (e.g. ggm or glasso)	Applied to metabolomics data by Krumsiek *et al*. (2011)
Mixed graphical models (MGMs)	–	Binary, continuous and mixed data		–
Bayesian Networks	–	Binary, continuous and mixed data	Several R packages (e.g. bnlearn, gRain, abn, deal)	Applied to transcriptomics data by Friedman *et al*. (2000)

The methods presented here are just a selection of the available methods for graph inference. Several other methods such as Boolean networks (Shmulevich *et al*., 2002) or differential equation systems (Chen *et al*., 1999; Lorenz *et al*., 2009) are commonly used for modelling biological networks.

The development of new techniques facilitates graph inference from high‐dimensional data, and the presented studies illustrate their usefulness in biological research. However, most graph inference methods rely on large sample sizes and usually more samples than variables are needed. When analysing omics data, particularly genomics or transcriptomics, this is often not feasible and it is referred to as the *n≪p* problem. Another common problem is overfitting of models due to the high number of parameters. Some techniques such as regularization have been proposed to relax these constraints and reduce overfitting. Nevertheless, stringent cross‐validation and replication in independent cohorts should be employed to avoid spurious results. Finally, many high‐throughput methods suffer from considerable technical variation and strong batch effects. Researchers should carefully normalize all measurements according to current standards before integrating different data sets.

### Model biological systems

The ultimate goal of systems biology is not only the qualitative exploration, but the quantitative modelling of the organism, facilitating *in silico* experiments, hypotheses generation and predictions.

The first – and so far only ‐ attempt to model a whole organism was conducted by Karr *et al*. (2012). They created a model of a mycoplasma genitalium cell simulating cell cycle and predicting metabolite concentrations. However, the model is far from perfect (Freddolino & Tavazoie, 2012) and too primitive to be adapted to more complex organisms. Currently, modelling eukaryotic cells or even whole organisms is not feasible. Also, processes like aging are too complex to be entirely modelled. However, some effort has been undertaken to create network representations of smaller subsystems as well as certain aspects of the aging process. For instance, Gillespie *et al*. (2004) simulated aging of yeast based on the accumulation of extrachromosomal ribosomal DNA circles. Also, Oda & Kitano (2006) summarized results from several hundred studies to create a model of the Toll‐like receptor (TLR) signalling network. The same group also created a similar model for epidermal growth factor receptor signalling (Oda *et al*., 2005). Both studies revealed a bowtie‐like global structure with one important key regulator. However, both networks are only qualitative descriptions without kinetic parameters. Thus, they cannot be used for computer simulations.

Other groups concentrated on even smaller subsystems to facilitate quantitative modelling. One study investigated the influence of increased cortisol levels on hippocampus activity (McAuley *et al*., 2009). A quantitative model was created to simulate the decline in hippocampal output with age and the acceleration of this process due to acute and chronic increases in cortisol levels. Simulations using ordinary differential equations suggested that chronic increase in cortisol levels leads to faster decline in hippocampal output than acute bursts, but could be treated more efficiently. Sozou & Kirkwood (2001) modelled cell senescence based on telomere shortening and oxidative stress. The same group also described the influence of chaperones and accumulation of misfolded proteins on aging (Proctor *et al*., 2005). Other groups investigated various further aspects of the aging process, such as mitochondrial fusion and fission events and accumulation of defective mitochondria (Kowald *et al*., 2005; Figge *et al*., 2012), incomplete replication of epigenetic information (Przybilla *et al*., 2014) and age‐related alterations in the lipid metabolism (McAuley & Mooney, 2015). Adjusting the kinetics of such models to correspond to experimental observations allows to come up with plausible hypotheses about the causes of aging.

In contrast to earlier presented networks, which inferred large‐scale networks from data (top‐down approach), these approaches model small subsystems in high details based on expert *a priori* knowledge (bottom‐up approach). Such bottom‐up models allow mechanistic insights into the processes of aging that cannot be generated by individual association studies. Moreover, they facilitate the development of new hypothesis and testing the plausibility of current hypothesis.

## Conclusions and challenges

The major recent advances of omics technologies are now enabling the simultaneous measurement of millions of biochemical entities. Association studies have revealed many associations of omics data with aging and age‐related diseases. After decades of reductionist studies, network analysis and integrated omics data analysis have begun to target the aging process at a systems level. As a result, some studies take into account also the interaction effects between variables. However, given the complexity of aging, new methods are needed to further unveil the multiple interactions.

Systems biology already provides such methods, but their application on real biological problems lags behind. For example, GGMs have been adapted to mixed data types and could readily be applied in aging research. Also, several studies developed models of processes that contribute to aging. These provide detailed knowledge about important components of the aging process and their interactions. Building on these results, future studies should aim to integrate these different parts to gain a more systems‐level understanding of aging.

However, in many cases, the available data limit the possibilities. Problems such as incomplete data, asynchronous experiments, strong batch effects and insufficient sample sizes have to be dealt with. Another issue is the limited availability of multi‐omics data sets, which complicates replication of results in this field. A variety of different methods, protocols and platforms further hampers reproducible results. As replication of results is crucial to prevent spurious results and validation, methods like splitting the available data into discovery and replication sets should be considered more often.

Despite these obstacles, there are several large population studies in existence with multi‐omics data available which could be explored using systems biology approaches. For instance, the GTEx project aims to collect gene expression and methylation data from multitissue samples (The Gtex Consortium 2013). Simultaneously, the development of new methods should help to analyse real, partially incomplete data sets and facilitate analysis of multitissue and multi‐organ data, thus enabling the investigation of real systems‐level effects. Addressing these problems and developing integrated models of aging should improve our understanding of the aging process, thus allowing the development of strategies to improve health in old age.

## Funding

This work was supported by the EU Framework Programme 7 small‐scale focused research collaborative project EurHEALTHAging [277849]; TwinsUK was funded by the Wellcome Trust; European Community's Seventh Framework Programme [FP7/2007‐2013]. The study also receives support from the National Institute for Health Research (NIHR) Clinical Research Facility at Guy's & St Thomas' NHS Foundation Trust and NIHR Biomedical Research Centre based at Guy's and St Thomas' NHS Foundation Trust and King's College London. TDS is a NIHR senior research fellow.

## Conflict of interest

None declared.

## References

[acel12386-bib-0001] Adamczyk B , Tharmalingam T , Rudd PM (2012) Glycans as cancer biomarkers. Biochim. Biophys. Acta 1820, 1347–1353. 22178561 10.1016/j.bbagen.2011.12.001

[acel12386-bib-0002] Albert R , Jeong H , Barabazi A‐L (2000) Error and attack tolerance of complex networks. Nature 406, 378–382. 10935628 10.1038/35019019

[acel12386-bib-0003] Alegría‐Torres JA , Baccarelli A , Bollati V (2011) Epigenetics and lifestyle. Epigenomics 3, 267–277. 22122337 10.2217/epi.11.22 PMC3752894

[acel12386-bib-0004] Allocco DJ , Kohane IS , Butte AJ (2004) Quantifying the relationship between co‐expression, co‐regulation and gene function. BMC Bioinformatics 5, 18. 15053845 10.1186/1471-2105-5-18 PMC375525

[acel12386-bib-0005] Alzheimer's Society (2014) Demography. Available at: http://www.alzheimers.org.uk.

[acel12386-bib-0006] Ashburner M , Ball CA , Blake JA , Botstein D , Butler H , Cherry JM , Davis AP , Dolinski K , Dwight SS , Eppig JT , Harris MA , Hill DP , Issel‐Tarver L , Kasarskis A , Lewis S , Matese JC , Richardson JE , Ringwald M , Rubin GM , Sherlock G (2000) Gene ontology: tool for the unification of biology. The gene ontology consortium. Nat. Genet. 25, 25–29. 10802651 10.1038/75556 PMC3037419

[acel12386-bib-0007] d'Aspremont A , Banerjee O , El Ghaoui L (2006) First‐order methods for sparse covariance selection. J. Mach. Learn. Res. 10, 883–906.

[acel12386-bib-0008] Bacalini MG , Friso S , Olivieri F , Pirazzini C , Giuliani C , Capri M , Santoro A , Franceschi C , Garagnani P (2014) Present and future of anti‐ageing epigenetic diets. Mech. Ageing Dev. 136–137, 101–115. 10.1016/j.mad.2013.12.006 24388875

[acel12386-bib-0009] Barabási A‐L , Oltvai ZN (2004) Network biology: understanding the cell's functional organization. Nat. Rev. Genet. 5, 101–113. 14735121 10.1038/nrg1272

[acel12386-bib-0010] Baylis D , Ntani G , Edwards MH , Syddall HE , Bartlett DB , Dennison EM , Martin‐Ruiz C , von Zglinicki T , Kuh D , Lord JM , Aihie Sayer A , Cooper C (2014) Inflammation, telomere length, and grip strength: a 10‐year longitudinal study. Calcif. Tissue Int. 95, 54–63. 24858709 10.1007/s00223-014-9862-7 PMC4098723

[acel12386-bib-0011] Beisser D , Klau GW , Dandekar T , Müller T , Dittrich MT (2010) BioNet: an R‐Package for the functional analysis of biological networks. Bioinformatics 26, 1129–1130. 20189939 10.1093/bioinformatics/btq089

[acel12386-bib-0012] Bell R , Hubbard A , Chettier R , Chen D , Miller JP , Kapahi P , Tarnopolsky M , Sahasrabuhde S , Melov S , Hughes RE (2009) A human protein interaction network shows conservation of aging processes between human and invertebrate species. PLoS Genet. 5, e1000414. 19293945 10.1371/journal.pgen.1000414 PMC2657003

[acel12386-bib-0013] Bell JT , Tsai P‐C , Yang T‐P , Pidsley R , Nisbet J , Glass D , Mangino M , Zhai G , Zhang F , Valdes A , Shin S‐Y , Dempster EL , Murray RM , Grundberg E , Hedman AK , Nica A , Small KS , Dermitzakis ET , McCarthy MI , Mill J , Spector TD , Deloukas P (2012) Epigenome‐wide scans identify differentially methylated regions for age and age‐related phenotypes in a healthy ageing population. PLoS Genet. 8, e1002629. 22532803 10.1371/journal.pgen.1002629 PMC3330116

[acel12386-bib-0014] Berchtold NC , Cribbs DH , Coleman PD , Rogers J , Head E , Kim R , Beach T , Miller C , Troncoso J , Trojanowski JQ , Zielke HR , Cotman CW (2008) Gene expression changes in the course of normal brain aging are sexually dimorphic. Proc. Natl Acad. Sci. U. S. A. 105, 15605–15610. 18832152 10.1073/pnas.0806883105 PMC2563070

[acel12386-bib-0015] Biagi E , Nylund L , Candela M , Ostan R , Bucci L , Pini E , Nikkïla J , Monti D , Satokari R , Franceschi C , Brigidi P , De Vos W (2010) Through ageing, and beyond: gut microbiota and inflammatory status in seniors and centenarians. PLoS ONE 5, e10667. 20498852 10.1371/journal.pone.0010667 PMC2871786

[acel12386-bib-0016] Biagi E , Candela M , Fairweather‐Tait S , Franceschi C , Brigidi P (2012) Aging of the human metaorganism: the microbial counterpart. Age (Dordr). 34, 247–267. 21347607 10.1007/s11357-011-9217-5 PMC3260362

[acel12386-bib-0017] Breitling LP , Yang R , Korn B , Burwinkel B , Brenner H (2011) Tobacco‐smoking‐related differential DNA methylation: 27K discovery and replication. Am. J. Hum. Genet. 88, 450–457. 21457905 10.1016/j.ajhg.2011.03.003 PMC3071918

[acel12386-bib-0018] Brion MA , Benyamin B , Visscher PM , Smith GD (2014) Beyond the single SNP: emerging developments in mendelian randomization in the ‘omics ‘era. Curr. Epidemiol. Reports 1, 228–236.

[acel12386-bib-0019] Cassman M (2005) Barriers to progress in systems biology. Nature 438, 1079. 16371982 10.1038/4381079a

[acel12386-bib-0020] Cevenini E , Invidia L , Lescai F , Salvioli S , Tieri P , Castellani G , Franceschi C (2008) Human models of aging and longevity. Expert Opin. Biol. Ther. 8, 1393–1405. 18694357 10.1517/14712598.8.9.1393

[acel12386-bib-0021] Chatr‐Aryamontri A , Breitkreutz B‐J , Heinicke S , Boucher L , Winter A , Stark C , Nixon J , Ramage L , Kolas N , O'Donnell L , Reguly T , Breitkreutz A , Sellam A , Chen D , Chang C , Rust J , Livstone M , Oughtred R , Dolinski K , Tyers M (2013) The BioGRID interaction database: 2013 update. Nucleic Acids Res. 41, D816–D823. 23203989 10.1093/nar/gks1158 PMC3531226

[acel12386-bib-0022] Chen T , He HL , Church GM (1999) Modeling gene expression with differential equations. Pac. Symp. Biocomput. 4, 29–40. 10380183

[acel12386-bib-0023] Chen S , Witten D , Shojaie A (2013) Selection and Estimation for Mixed Graphical Models. Available at: http://arxiv.org/abs/1311.0085 [Accessed January 7, 2014]. 10.1093/biomet/asu051 PMC5018402 27625437

[acel12386-bib-0024] Claesson MJ , Cusack S , O'Sullivan O , Greene‐Diniz R , de Weerd H , Flannery E , Marchesi JR , Falush D , Dinan T , Fitzgerald G , Stanton C , van Sinderen D , O'Connor M , Harnedy N , O'Connor K , Henry C , O'Mahony D , Fitzgerald AP , Shanahan F , Twomey C , Hill C , Ross RP , O'Toole PW (2011) Composition, variability, and temporal stability of the intestinal microbiota of the elderly. Proc. Natl Acad. Sci. U. S. A. 108(Suppl), 4586–4591. 20571116 10.1073/pnas.1000097107 PMC3063589

[acel12386-bib-0025] Claesson MJ , Jeffery IB , Conde S , Power SE , O'Connor EM , Cusack S , Harris HMB , Coakley M , Lakshminarayanan B , O'Sullivan O , Fitzgerald GF , Deane J , O'Connor M , Harnedy N , O'Connor K , O'Mahony D , van Sinderen D , Wallace M , Brennan L , Stanton C , Marchesi JR , Fitzgerald AP , Shanahan F , Hill C , Ross RP , O'Toole PW (2012) Gut microbiota composition correlates with diet and health in the elderly. Nature 488, 178–184. 22797518 10.1038/nature11319

[acel12386-bib-0026] Craig T , Smelick C , Tacutu R , Wuttke D , Wood SH , Stanley H , Janssens G , Savitskaya E , Moskalev A , Arking R , de Magalhaes JP (2014) The Digital Ageing Atlas: integrating the diversity of age‐related changes into a unified resource. Nucleic Acids Res. 43, D873–D878. 25232097 10.1093/nar/gku843 PMC4384002

[acel12386-bib-0027] Croft D , Mundo AF , Haw R , Milacic M , Weiser J , Wu G , Caudy M , Garapati P , Gillespie M , Kamdar MR , Jassal B , Jupe S , Matthews L , May B , Palatnik S , Rothfels K , Shamovsky V , Song H , Williams M , Birney E , Hermjakob H , Stein L , D'Eustachio P (2014) The reactome pathway knowledgebase. Nucleic Acids Res. 42, D472–D477. 24243840 10.1093/nar/gkt1102 PMC3965010

[acel12386-bib-0028] Csardi G , Nepusz T (2006) The igraph software package for complex network research. InterJournal Complex Sy, 1695, Available at: http://igraph.org.

[acel12386-bib-0029] Dang W , Steffen KK , Perry R , Dorsey JA , Johnson FB , Shilatifard A , Kaeberlein M , Kennedy BK , Berger SL (2009) Histone H4 lysine 16 acetylation regulates cellular lifespan. Nature 459, 802–807. 19516333 10.1038/nature08085 PMC2702157

[acel12386-bib-0030] Davies G , Harris SE , Reynolds CA , Payton A , Knight HM , Liewald DC , Lopez LM , Luciano M , Gow AJ , Corley J , Henderson R , Murray C , Pattie A , Fox HC , Redmond P , Lutz MW , Chiba‐Falek O , Linnertz C , Saith S , Haggarty P , McNeill G , Ke X , Ollier W , Horan M , Roses AD , Ponting CP , Porteous DJ , Tenesa A , Pickles A , Starr JM , Whalley LJ , Pedersen NL , Pendleton N , Visscher PM , Deary IJ (2014) A genome‐wide association study implicates the APOE locus in nonpathological cognitive ageing. Mol. Psychiatry 19, 76–87. 23207651 10.1038/mp.2012.159 PMC7321835

[acel12386-bib-0031] Deelen J , Beekman M , Uh HW , Helmer Q , Kuningas M , Christiansen L , Kremer D , van der Breggen R , Suchiman HED , Lakenberg N , van den Akker EB , Passtoors WM , Tiemeier H , van Heemst D , de Craen AJ , Rivadeneira F , de Geus EJ , Perola M , van der Ouderaa FJ , Gunn DA , Boomsma DI , Uitterlinden AG , Christensen K , van Duijn CM , Heijmans BT , Houwing‐Duistermaat JJ , Westendorp RGJ , Slagboom PE (2011) Genome‐wide association study identifies a single major locus contributing to survival into old age; the APOE locus revisited. Aging Cell 10, 686–698. 21418511 10.1111/j.1474-9726.2011.00705.x PMC3193372

[acel12386-bib-0032] Eddy SR (2001) Non‐coding RNA genes and the modern RNA world. Nat. Rev. Genet. 2, 919–929. 11733745 10.1038/35103511

[acel12386-bib-0033] Ehrlich M (2002) DNA methylation in cancer: too much, but also too little. Oncogene 21, 5400–5413. 12154403 10.1038/sj.onc.1205651

[acel12386-bib-0034] Faisal FE , Milenković T (2014) Dynamic networks reveal key players in aging. Bioinformatics 30, 1721–1729. 24554629 10.1093/bioinformatics/btu089

[acel12386-bib-0035] Fellinghauer B , Bühlmann P , Ryffel M , von Rhein M , Reinhardt JD (2013) Stable graphical model estimation with Random Forests for discrete, continuous, and mixed variables. Comput. Stat. Data Anal. 64, 132–152.

[acel12386-bib-0036] Figge MT , Reichert AS , Meyer‐Hermann M , Osiewacz HD (2012) Deceleration of fusion‐fission cycles improves mitochondrial quality control during aging. PLoS Comput. Biol. 8, e1002576. 22761564 10.1371/journal.pcbi.1002576 PMC3386171

[acel12386-bib-0037] Flachsbart F , Caliebe A , Kleindorp R , Blanché H , von Eller‐Eberstein H , Nikolaus S , Schreiber S , Nebel A (2009) Association of FOXO3A variation with human longevity confirmed in German centenarians. Proc. Natl Acad. Sci. U. S. A. 106, 2700–2705. 19196970 10.1073/pnas.0809594106 PMC2650329

[acel12386-bib-0038] Franceschini A , Szklarczyk D , Frankild S , Kuhn M , Simonovic M , Roth A , Lin J , Minguez P , Bork P , von Mering C , Jensen LJ (2013) STRING v9.1: protein‐protein interaction networks, with increased coverage and integration. Nucleic Acids Res. 41, D808–D815. 23203871 10.1093/nar/gks1094 PMC3531103

[acel12386-bib-0039] Freddolino PL , Tavazoie S (2012) The dawn of virtual cell biology. Cell 150, 248–250. 22817888 10.1016/j.cell.2012.07.001 PMC4430847

[acel12386-bib-0040] Friedman N , Linial M , Nachman I , Pe'er D (2000) Using Bayesian networks to analyze expression data. J. Comput. Biol. 7, 601–620. 11108481 10.1089/106652700750050961

[acel12386-bib-0041] Friedman J , Hastie T , Tibshirani R (2008) Sparse inverse covariance estimation with the graphical lasso. Biostatistics 9, 432–441. 18079126 10.1093/biostatistics/kxm045 PMC3019769

[acel12386-bib-0042] de la Fuente A , Bing N , Hoeschele I , Mendes P (2004) Discovery of meaningful associations in genomic data using partial correlation coefficients. Bioinformatics 20, 3565–3574. 15284096 10.1093/bioinformatics/bth445

[acel12386-bib-0043] Fuster MM , Esko JD (2005) The sweet and sour of cancer: glycans as novel therapeutic targets. Nat. Rev. Cancer 5, 526–542. 16069816 10.1038/nrc1649

[acel12386-bib-0044] Gatz M , Reynolds CA , Fratiglioni L , Johansson B , Mortimer JA , Berg S , Fiske A , Pedersen NL (2006) Role of genes and environments for explaining Alzheimer disease. Arch. Gen. Psychiatry 63, 168–174. 16461860 10.1001/archpsyc.63.2.168

[acel12386-bib-0045] Gelfi C , Vigano A , Ripamonti M , Pontoglio A , Begum S , Pellegrino MA , Grassi B , Bottinelli R , Wait R , Cerretelli P (2006) The human muscle proteome in aging. J. Proteome Res. 5, 1344–1353. 16739986 10.1021/pr050414x

[acel12386-bib-0046] Gillespie CS , Proctor CJ , Boys RJ , Shanley DP , Wilkinson DJ , Kirkwood TBL (2004) A mathematical model of ageing in yeast. J. Theor. Biol. 229, 189–196. 15207474 10.1016/j.jtbi.2004.03.015

[acel12386-bib-0047] Ginsburg GS , Haga SB (2006) Translating genomic biomarkers into clinically useful diagnostics. Expert Rev. Mol. Diagn. 6, 179–191. 16512778 10.1586/14737159.6.2.179

[acel12386-bib-0048] Glass D , Viñuela A , Davies MN , Ramasamy A , Parts L , Knowles D , Brown AA , Hedman AK , Small KS , Buil A , Grundberg E , Nica AC , Meglio P , Nestle FO , Ryten M , Durbin R , McCarthy MI , Deloukas P , Dermitzakis ET , Weale ME , Bataille V , Spector TD (2013) Gene expression changes with age in skin, adipose tissue, blood and brain. Genome Biol. 14, R75. 23889843 10.1186/gb-2013-14-7-r75 PMC4054017

[acel12386-bib-0049] Goh K , Cusick ME , Valle D , Childs B , Vidal M , Barabási A‐L (2007) The human disease network. Proc. Natl Acad. Sci. U. S. A. 104, 8685–8690. 17502601 10.1073/pnas.0701361104 PMC1885563

[acel12386-bib-0050] Goodrich JK , Waters JL , Poole AC , Sutter JL , Koren O , Blekhman R , Beaumont M , Van Treuren W , Knight R , Bell JT , Spector TD , Clark AG , Ley RE (2014) Human genetics shape the gut microbiome. Cell 159, 789–799. 25417156 10.1016/j.cell.2014.09.053 PMC4255478

[acel12386-bib-0051] Gornik O , Wagner J , Pucić M , Knezević A , Redzic I , Lauc G (2009) Stability of N‐glycan profiles in human plasma. Glycobiology 19, 1547–1553. 19726492 10.1093/glycob/cwp134

[acel12386-bib-0052] Greer EL , Maures TJ , Hauswirth AG , Green EM , Leeman DS , Maro GS , Han S , Banko MR , Gozani O , Brunet A (2010) Members of the H3K4 trimethylation complex regulate lifespan in a germline‐dependent manner in *C. elegans* . Nature 466, 383–387. 20555324 10.1038/nature09195 PMC3075006

[acel12386-bib-0053] Grice EA , Segre JA (2012) The human microbiome: our second genome. Annu. Rev. Genomics Hum. Genet. 13, 151–170. 22703178 10.1146/annurev-genom-090711-163814 PMC3518434

[acel12386-bib-0054] Guigoz Y , Doré J , Schiffrin EJ (2008) The inflammatory status of old age can be nurtured from the intestinal environment. Curr. Opin. Clin. Nutr. Metab. Care 11, 13–20. 18090652 10.1097/MCO.0b013e3282f2bfdf

[acel12386-bib-0055] Guo J , Levina E , Michailidis G , Zhu J (2010) Joint Structure Estimation for Categorical Markov Networks.

[acel12386-bib-0056] Ha N‐T , Freytag S , Bickeboeller H (2014) Coverage and efficiency in current SNP chips. Eur. J. Hum. Genet. 22, 1124–1130. 24448550 10.1038/ejhg.2013.304 PMC4135415

[acel12386-bib-0057] Hammond CJ , Duncan DD , Snieder H , de Lange M , West SK , Spector TD , Gilbert CE (2001) The heritability of age‐related cortical cataract: the twin eye study. Invest. Ophthalmol. Vis. Sci. 42, 601–605. 11222516

[acel12386-bib-0058] Harman D (1988) The aging process. Basic Life Sci. 49, 1057–1065. 3250468 10.1007/978-1-4684-5568-7_175

[acel12386-bib-0059] Harman D (2001) Aging: overview. Ann. N. Y. Acad. Sci. 928, 1–21. 11795501 10.1111/j.1749-6632.2001.tb05631.x

[acel12386-bib-0060] Höfling H , Tibshirani R (2009) Estimation of sparse binary pairwise markov networks using pseudo‐likelihoods. J. Mach. Learn. Res. 10, 883–906. 21857799 PMC3157941

[acel12386-bib-0061] Horvath S (2013) DNA methylation age of human tissues and cell types. Genome Biol. 14, R115. 24138928 10.1186/gb-2013-14-10-r115 PMC4015143

[acel12386-bib-0062] Houle D , Govindaraju DR , Omholt S (2010) Phenomics: the next challenge. Nat. Rev. Genet. 11, 855–866. 21085204 10.1038/nrg2897

[acel12386-bib-0063] Houseman E , Accomando WP , Koestler DC , Christensen BC , Marsit CJ , Nelson HH , Wiencke JK , Kelsey KT (2012) DNA methylation arrays as surrogate measures of cell mixture distribution. BMC Bioinformatics 13, 86. 22568884 10.1186/1471-2105-13-86 PMC3532182

[acel12386-bib-0064] Huang H , Bader JS (2009) Precision and recall estimates for two‐hybrid screens. Bioinformatics 25, 372–378. 19091773 10.1093/bioinformatics/btn640 PMC2639075

[acel12386-bib-0065] Ideker T , Galitski T , Hood L (2001) A new approach to decoding life: systems biology. Annu. Rev. Genomics Hum. Genet. 2, 343–372. 11701654 10.1146/annurev.genom.2.1.343

[acel12386-bib-0066] Ideker T , Ozier O , Schwikowski B , Siegel AF (2002) Discovering regulatory and signalling circuits in molecular interaction networks. Bioinformatics 18(Suppl 1), S233–S240. 12169552 10.1093/bioinformatics/18.suppl_1.s233

[acel12386-bib-0067] Illumnia (2011) Infinium HumanMethylation450 BeadChip Kit. Available at: http://www.illumina.com/products/methylation_450_beadchip_kits.ilmn [Accessed June 11, 2014].

[acel12386-bib-0068] Ishimori ML , Altman RD , Cohen MJ , Cui J , Guo X , Rotter JI , Weisman MH (2010) Heritability patterns in hand osteoarthritis: the role of osteophytes. Arthritis. Res. Ther. 12, R180. 20920163 10.1186/ar3144 PMC2991011

[acel12386-bib-0069] Jackson SHD , Weale MR , Weale RA (2003) Biological age–what is it and can it be measured? Arch. Gerontol. Geriatr. 36, 103–115. 12849085 10.1016/s0167-4943(02)00060-2

[acel12386-bib-0070] Jaenisch R , Bird A (2003) Epigenetic regulation of gene expression: how the genome integrates intrinsic and environmental signals. Nat. Genet. 33(Suppl), 245–254. 12610534 10.1038/ng1089

[acel12386-bib-0071] Jaffe AE , Irizarry RA (2014) Accounting for cellular heterogeneity is critical in epigenome‐wide association studies. Genome Biol. 15, R31. 24495553 10.1186/gb-2014-15-2-r31 PMC4053810

[acel12386-bib-0072] Jeong H , Mason SP , Barabási AL , Oltvai ZN (2001) Lethality and centrality in protein networks. Nature 411, 41–42. 11333967 10.1038/35075138

[acel12386-bib-0073] Johnson TE (2006) Recent results: biomarkers of aging. Exp. Gerontol. 41, 1243–1246. 17071038 10.1016/j.exger.2006.09.006

[acel12386-bib-0074] Joy MP , Brock A , Ingber DE , Huang S (2005) High‐betweenness proteins in the yeast protein interaction network. J. Biomed. Biotechnol. 2005, 96–103. 16046814 10.1155/JBB.2005.96 PMC1184047

[acel12386-bib-0075] Kanehisa M , Goto S (2000) KEGG: kyoto encyclopedia of genes and genomes. Nucleic Acids Res. 28, 27–30. 10592173 10.1093/nar/28.1.27 PMC102409

[acel12386-bib-0076] Karr JR , Sanghvi JC , Macklin DN , Gutschow MV , Jacobs JM , Bolival B , Assad‐Garcia N , Glass JI , Covert MW (2012) A whole‐cell computational model predicts phenotype from genotype. Cell 150, 389–401. 22817898 10.1016/j.cell.2012.05.044 PMC3413483

[acel12386-bib-0077] Keshava Prasad TS , Goel R , Kandasamy K , Keerthikumar S , Kumar S , Mathivanan S , Telikicherla D , Raju R , Shafreen B , Venugopal A , Balakrishnan L , Marimuthu A , Banerjee S , Somanathan DS , Sebastian A , Rani S , Ray S , Harrys Kishore CJ , Kanth S , Ahmed M , Kashyap MK , Mohmood R , Ramachandra YL , Krishna V , Rahiman BA , Mohan S , Ranganathan P , Ramabadran S , Chaerkady R , Pandey A (2009) Human protein reference database–2009 update. Nucleic Acids Res. 37, D767–D772. 18988627 10.1093/nar/gkn892 PMC2686490

[acel12386-bib-0078] Kirkwood TB , Austad SN (2000) Why do we age? Nature 408, 233–238. 11089980 10.1038/35041682

[acel12386-bib-0079] Knezević A , Polasek O , Gornik O , Rudan I , Campbell H , Hayward C , Wright A , Kolcic I , O'Donoghue N , Bones J , Rudd PM , Lauc G (2009) Variability, heritability and environmental determinants of human plasma N‐glycome. J. Proteome Res. 8, 694–701. 19035662 10.1021/pr800737u

[acel12386-bib-0080] Kong HH (2011) Skin microbiome: genomics‐based insights into the diversity and role of skin microbes. Trends Mol. Med. 17, 320–328. 21376666 10.1016/j.molmed.2011.01.013 PMC3115422

[acel12386-bib-0081] Koropatkin NM , Cameron EA , Martens EC (2012) How glycan metabolism shapes the human gut microbiota. Nat. Rev. Microbiol. 10, 323–335. 22491358 10.1038/nrmicro2746 PMC4005082

[acel12386-bib-0082] Kowald A , Jendrach M , Pohl S , Bereiter‐Hahn J , Hammerstein P (2005) On the relevance of mitochondrial fusions for the accumulation of mitochondrial deletion mutants: a modelling study. Aging Cell 4, 273–283. 16164426 10.1111/j.1474-9726.2005.00169.x

[acel12386-bib-0083] Kriete A , Lechner M , Clearfield D , Bohmann D (2011) Computational systems biology of aging. Wiley Interdiscip. Rev. Syst. Biol. Med. 3, 414–428. 21197651 10.1002/wsbm.126

[acel12386-bib-0084] Kristic J , Vuckovic F , Menni C , Klaric L , Keser T , Beceheli I , Pucic‐Bakovic M , Novokmet M , Mangino M , Thaqi K , Rudan P , Novokmet N , Sarac J , Missoni S , Kolcic I , Polasek O , Rudan I , Campbell H , Hayward C , Aulchenko Y , Valdes A , Wilson JF , Gornik O , Primorac D , Zoldos V , Spector T , Lauc G (2013) Glycans are a novel biomarker of chronological and biological ages. J. Gerontol. A Biol. Sci. Med. Sci., 69, 1–11. 24325898 10.1093/gerona/glt190 PMC4049143

[acel12386-bib-0085] Krumsiek J , Suhre K , Illig T , Adamski J , Theis FJ (2011) Gaussian graphical modeling reconstructs pathway reactions from high‐throughput metabolomics data. BMC Syst. Biol. 5, 21. 21281499 10.1186/1752-0509-5-21 PMC3224437

[acel12386-bib-0086] Lawton KA , Berger A , Mitchell M , Milgram KE , Evans AM , Guo L , Hanson RW , Kalhan SC , Ryals JA , Milburn MV (2008) Analysis of the adult human plasma metabolome. Pharmacogenomics 9, 383–397. 18384253 10.2217/14622416.9.4.383

[acel12386-bib-0087] Lee JD , Hastie TJ (2015) Learning the structure of mixed graphical models. J. Comput. Graph. Stat. 24, 230–253. 26085782 10.1080/10618600.2014.900500 PMC4465824

[acel12386-bib-0088] Levine ME (2013) Modeling the rate of senescence: can estimated biological age predict mortality more accurately than chronological age? J. Gerontol. A Biol. Sci. Med. Sci. 68, 667–674. 23213031 10.1093/gerona/gls233 PMC3660119

[acel12386-bib-0089] Liu L , Li Y , Li S , Hu N , He Y , Pong R , Lin D , Lu L , Law M (2012) Comparison of next‐generation sequencing systems. J. Biomed. Biotechnol. 2012, 1–11. 22829749 10.1155/2012/251364 PMC3398667

[acel12386-bib-0090] Lodish HF (2013) Molecular Cell Biology, 7th edn New York: W.H. Freeman and Co.

[acel12386-bib-0091] López‐Otín C , Blasco MA , Partridge L , Serrano M , Kroemer G (2013) The hallmarks of aging. Cell 153, 1194–1217. 23746838 10.1016/j.cell.2013.05.039 PMC3836174

[acel12386-bib-0092] Lorenz DR , Cantor CR , Collins JJ (2009) A network biology approach to aging in yeast. Proc. Natl Acad. Sci. U. S. A. 106, 1145–1150. 19164565 10.1073/pnas.0812551106 PMC2629491

[acel12386-bib-0093] Lu T , Pan Y , Kao S , Li C , Kohane I , Chan J , Yankner BA (2004) Gene regulation and DNA damage in the ageing human brain. Nature 429, 883–891. 15190254 10.1038/nature02661

[acel12386-bib-0094] Luo W , Friedman MS , Shedden K , Hankenson KD , Woolf PJ (2009) GAGE: generally applicable gene set enrichment for pathway analysis. BMC Bioinformatics 10, 161. 19473525 10.1186/1471-2105-10-161 PMC2696452

[acel12386-bib-0095] de Magalhães JP , Curado J , Church GM (2009) Meta‐analysis of age‐related gene expression profiles identifies common signatures of aging. Bioinformatics 25, 875–881. 19189975 10.1093/bioinformatics/btp073 PMC2732303

[acel12386-bib-0096] Mangin B , Siberchicot A , Nicolas S , Doligez A , This P , Cierco‐Ayrolles C (2012) Novel measures of linkage disequilibrium that correct the bias due to population structure and relatedness. Heredity (Edinb). 108, 285–291. 21878986 10.1038/hdy.2011.73 PMC3282397

[acel12386-bib-0097] Marbach D , Costello JC , Küffner R , Vega NM , Prill RJ , Camacho DM , Allison KR , Kellis M , Collins JJ , Stolovitzky G (2012) Wisdom of crowds for robust gene network inference. Nat. Methods 9, 796–804. 22796662 10.1038/nmeth.2016 PMC3512113

[acel12386-bib-0098] Mazumder R , Hastie T (2012) The graphical lasso: new insights and alternatives. Electron. J. Stat. 6, 2125–2149. 25558297 10.1214/12-EJS740 PMC4281944

[acel12386-bib-0099] McAuley MT , Mooney KM (2015) Computationally modeling lipid metabolism and aging: a mini‐review. Comput. Struct. Biotechnol. J. 13, 38–46. 25750699 10.1016/j.csbj.2014.11.006 PMC4348429

[acel12386-bib-0100] McAuley MT , Kenny RA , Kirkwood TBL , Wilkinson DJ , Jones JJL , Miller VM (2009) A mathematical model of aging‐related and cortisol induced hippocampal dysfunction. BMC Neurosci. 10, 26. 19320982 10.1186/1471-2202-10-26 PMC2680862

[acel12386-bib-0101] Mehra VC , Ramgolam VS , Bender JR (2005) Cytokines and cardiovascular disease. J. Leukoc. Biol. 78, 805–818. 16006537 10.1189/jlb.0405182

[acel12386-bib-0102] Meinshausen N , Bühlmann P (2006) High‐dimensional graphs and variable selection with the Lasso. Ann. Stat. 34, 1436–1462.

[acel12386-bib-0103] Meinshausen N , Bühlmann P (2010) Stability selection. J. R. Stat. Soc. Ser. B (Statistical Methodol. 72, 417–473.

[acel12386-bib-0104] Meissner A (2010) Epigenetic modifications in pluripotent and differentiated cells. Nat. Biotechnol. 28, 1079–1088. 20944600 10.1038/nbt.1684

[acel12386-bib-0105] Menni C , Fauman E , Erte I , Perry JRB , Kastenmüller G , Shin SY , Petersen AK , Hyde C , Psatha M , Ward KJ , Yuan W , Milburn M , Palmer CNA , Frayling TM , Trimmer J , Bell JT , Gieger C , Mohney RP , Brosnan MJ , Suhre K , Soranzo N , Spector TD (2013a) Biomarkers for type 2 diabetes and impaired fasting glucose using a nontargeted metabolomics approach. Diabetes 62, 4270–4276. 23884885 10.2337/db13-0570 PMC3837024

[acel12386-bib-0106] Menni C , Kastenmüller G , Petersen AK , Bell JT , Psatha M , Tsai P‐C , Gieger C , Schulz H , Erte I , John S , Brosnan MJ , Wilson SG , Tsaprouni L , Lim EM , Stuckey B , Deloukas P , Mohney R , Suhre K , Spector TD , Valdes AM (2013b) Metabolomic markers reveal novel pathways of ageing and early development in human populations. Int. J. Epidemiol. 42, 1111–1119. 23838602 10.1093/ije/dyt094 PMC3781000

[acel12386-bib-0107] Menni C , Kiddle SJ , Mangino M , Vinuela A , Psatha M , Steves C , Sattlecker M , Buil A , Newhouse S , Nelson S , Williams S , Voyle N , Soininen H , Kloszewska I , Mecocci P , Tsolaki M , Vellas B , Lovestone S , Spector TD , Dobson R , Valdes AM (2015) Circulating proteomic signatures of chronological age. J. Gerontol. Ser. A Biol. Sci. Med. Sci. 70, 809–816. 25123647 10.1093/gerona/glu121 PMC4469006

[acel12386-bib-0108] Miller JA , Oldham MC , Geschwind DH (2008) A systems level analysis of transcriptional changes in Alzheimer's disease and normal aging. J. Neurosci. 28, 1410–1420. 18256261 10.1523/JNEUROSCI.4098-07.2008 PMC2902235

[acel12386-bib-0109] Mitnitski A , Song X , Rockwood K (2013) Assessing biological aging: the origin of deficit accumulation. Biogerontology 14, 709–717. 23860844 10.1007/s10522-013-9446-3 PMC3847281

[acel12386-bib-0110] Moayyeri A , Hammond CJ , Valdes AM , Spector TD (2013) Cohort profile: TwinsUK and healthy ageing twin study. Int. J. Epidemiol. 42, 76–85. 22253318 10.1093/ije/dyr207 PMC3600616

[acel12386-bib-0111] Mungreiphy NK , Kapoor S , Sinha R (2011) Association between BMI, blood pressure, and age: study among tangkhul naga tribal males of northeast India. J. Anthropol. 2011, 1–6.

[acel12386-bib-0112] Murabito JM , Yuan R , Lunetta KL (2012) The search for longevity and healthy aging genes: insights from epidemiological studies and samples of long‐lived individuals. J. Gerontol. A Biol. Sci. Med. Sci. 67, 470–479. 22499766 10.1093/gerona/gls089 PMC3326242

[acel12386-bib-0113] Nakajima K , Takeoka M , Mori M , Hashimoto S , Sakurai A , Nose H , Higuchi K , Itano N , Shiohara M , Oh T , Taniguchi S (2010) Exercise effects on methylation of ASC gene. Int. J. Sports Med. 31, 671–675. 20200803 10.1055/s-0029-1246140

[acel12386-bib-0114] Nebel A , Kleindorp R , Caliebe A , Nothnagel M , Blanché H , Junge O , Wittig M , Ellinghaus D , Flachsbart F , Wichmann HE , Meitinger T , Nikolaus S , Franke A , Krawczak M , Lathrop M , Schreiber S (2011) A genome‐wide association study confirms APOE as the major gene influencing survival in long‐lived individuals. Mech. Ageing Dev. 132, 324–330. 21740922 10.1016/j.mad.2011.06.008

[acel12386-bib-0115] Oda K , Kitano H (2006) A comprehensive map of the toll‐like receptor signaling network. Mol. Syst. Biol. 2, 2006.0015. 10.1038/msb4100057 PMC1681489 16738560

[acel12386-bib-0116] Oda K , Matsuoka Y , Funahashi A , Kitano H (2005) A comprehensive pathway map of epidermal growth factor receptor signaling. Mol. Syst. Biol. 1, 2005.0010. 10.1038/msb4100014 PMC1681468 16729045

[acel12386-bib-0117] Oeppen J , Vaupel JW (2002) Demography. Broken limits to life expectancy. Science 296, 1029–1031. 12004104 10.1126/science.1069675

[acel12386-bib-0118] Office for National Statistics (2014) Historic and Projected Data from the Period and Cohort Life Tables, 2012‐based revised. Available at: http://www.ons.gov.uk/ons/publications/re-reference-tables.html?edition=tcm:77-355125.

[acel12386-bib-0119] Orešič M , Hyötyläinen T , Herukka S‐K , Sysi‐Aho M , Mattila I , Seppänan‐Laakso T , Julkunen V , Gopalacharyulu PV , Hallikainen M , Koikkalainen J , Kivipelto M , Helisalmi S , Lötjönen J , Soininen H (2011) Metabolome in progression to Alzheimer's disease. Transl. Psychiatry 1, e57. 22832349 10.1038/tp.2011.55 PMC3309497

[acel12386-bib-0120] Pagel P , Kovac S , Oesterheld M , Brauner B , Dunger‐Kaltenbach I , Frishman G , Montrone C , Mark P , Stümpflen V , Mewes H‐W , Ruepp A , Frishman D (2005) The MIPS mammalian protein‐protein interaction database. Bioinformatics 21, 832–834. 15531608 10.1093/bioinformatics/bti115

[acel12386-bib-0121] Patti GJ , Yanes O , Siuzdak G (2012) Innovation: metabolomics: the apogee of the omics trilogy. Nat. Rev. Mol. Cell Biol. 13, 263–269. 22436749 10.1038/nrm3314 PMC3682684

[acel12386-bib-0122] Petersen AK , Zeilinger S , Kastenmüller G , Werner RM , Brugger M , Peters A , Meisinger C , Strauch K , Hengstenberg C , Pagel P , Huber F , Mohney RP , Grallert H , Illig T , Adamski J , Waldenberger M , Gieger C , Suhre K (2014) Epigenetics meets metabolomics: an epigenome‐wide association study with blood serum metabolic traits. Hum. Mol. Genet. 23, 534–545. 24014485 10.1093/hmg/ddt430 PMC3869358

[acel12386-bib-0123] Peterson J , Garges S , Giovanni M , McInnes P , Wang L , Schloss JA , Bonazzi V , McEwen JE , Wetterstrand KA , Deal C , Baker CC , Di Francesco V , Howcroft TK , Karp RW , Lunsford RD , Wellington CR , Belachew T , Wright M , Giblin C , David H , Mills M , Salomon R , Mullins C , Akolkar B , Begg L , Davis C , Grandison L , Humble M , Khalsa J , Little AR , Peavy H , Pontzer C , Portnoy M , Sayre MH , Starke‐Reed P , Zakhari S , Read J , Watson B , Guyer M (2009) The NIH human microbiome project. Genome Res. 19, 2317–2323. 19819907 10.1101/gr.096651.109 PMC2792171

[acel12386-bib-0124] Portela A , Esteller M (2010) Epigenetic modifications and human disease. Nat. Biotechnol. 28, 1057–1068. 20944598 10.1038/nbt.1685

[acel12386-bib-0125] Proctor CJ , Soti C , Boys RJ , Gillespie CS , Shanley DP , Wilkinson DJ , Kirkwood TBL (2005) Modelling the actions of chaperones and their role in ageing. Mech. Ageing Dev. 126, 119–131. 15610770 10.1016/j.mad.2004.09.031

[acel12386-bib-0126] Przybilla J , Rohlf T , Loeffler M , Galle J (2014) Understanding epigenetic changes in aging stem cells ‐ a computational model approach. Aging Cell 13, 320–328. 24428552 10.1111/acel.12177 PMC4331773

[acel12386-bib-0127] Pucić M , Knezević A , Vidic J , Adamczyk B , Novokmet M , Polasek O , Gornik O , Supraha‐Goreta S , Wormald MR , Redzić I , Campbell H , Wright A , Hastie ND , Wilson JF , Rudan I , Wuhrer M , Rudd PM , Josić D , Lauc G (2011) High throughput isolation and glycosylation analysis of IgG‐variability and heritability of the IgG glycome in three isolated human populations. Mol. Cell Proteomics 10, M111.010090. 10.1074/mcp.M111.010090 PMC3205872 21653738

[acel12386-bib-0128] Rattan SIS (2006) Theories of biological aging: genes, proteins, and free radicals. Free Radic. Res. 40, 1230–1238. 17090411 10.1080/10715760600911303

[acel12386-bib-0129] Ravikumar P , Wainwright MJ , Lafferty JD (2010) High‐dimensional Ising model selection using ℓ 1 ‐regularized logistic regression. Ann. Stat. 38, 1287–1319.

[acel12386-bib-0130] Reeve A , Simcox E , Turnbull D (2014) Ageing and Parkinson's disease: why is advancing age the biggest risk factor?. Ageing Res. Rev. 14C, 19–30. 24503004 10.1016/j.arr.2014.01.004 PMC3989046

[acel12386-bib-0131] Rockwood K , Mitnitski A (2007) Frailty in relation to the accumulation of deficits. J. Gerontol. Ser. A Biol. Sci. Med. Sci. 62, 722–727. 17634318 10.1093/gerona/62.7.722

[acel12386-bib-0132] Rodwell GEJ , Sonu R , Zahn JM , Lund J , Wilhelmy J , Wang L , Xiao W , Mindrinos M , Crane E , Segal E , Myers BD , Brooks JD , Davis RW , Higgins J , Owen AB , Kim SK (2004) A transcriptional profile of aging in the human kidney. PLoS Biol. 2, e427. 15562319 10.1371/journal.pbio.0020427 PMC532391

[acel12386-bib-0133] Round JL , Mazmanian SK (2009) The gut microbiota shapes intestinal immune responses during health and disease. Nat. Rev. Immunol. 9, 313–323. 19343057 10.1038/nri2515 PMC4095778

[acel12386-bib-0134] Royle L , Campbell MP , Radcliffe CM , White DM , Harvey DJ , Abrahams JL , Kim Y‐G , Henry GW , Shadick NA , Weinblatt ME , Lee DM , Rudd PM , Dwek RA (2008) HPLC‐based analysis of serum N‐glycans on a 96‐well plate platform with dedicated database software. Anal. Biochem. 376, 1–12. 18194658 10.1016/j.ab.2007.12.012

[acel12386-bib-0135] Scutari M (2010) Learning Bayesian networks with the bnlearn R package. J. Stat. Softw. 35, 1–22.21603108

[acel12386-bib-0136] Sebastiani P , Solovieff N , Dewan AT , Walsh KM , Puca A , Hartley SW , Melista E , Andersen S , Dworkis DA , Wilk JB , Myers RH , Steinberg MH , Montano M , Baldwin CT , Hoh J , Perls TT (2012) Genetic signatures of exceptional longevity in humans. PLoS ONE 7, e29848. 22279548 10.1371/journal.pone.0029848 PMC3261167

[acel12386-bib-0137] Shannon P , Markiel A , Ozier O , Baliga NS , Wang JT , Ramage D , Amin N , Schwikowski B , Ideker T (2003) Cytoscape: a software environment for integrated models of biomolecular interaction networks. Genome Res. 13, 2498–2504. 14597658 10.1101/gr.1239303 PMC403769

[acel12386-bib-0138] Shin S‐Y , Fauman EB , Petersen A‐K , Krumsiek J , Santos R , Huang J , Arnold M , Erte I , Forgetta V , Yang T‐P , Walter K , Menni C , Chen L , Vasquez L , Valdes AM , Hyde CL , Wang V , Ziemek D , Roberts P , Xi L , Grundberg E , Waldenberger M , Richards JB , Mohney RP , Milburn MV , John SL , Trimmer J , Theis FJ , Overington JP , Suhre K , Brosnan MJ , Gieger C , Kastenmüller G , Spector TD , Soranzo N (2014) An atlas of genetic influences on human blood metabolites. Nat. Genet. 46, 543–550. 24816252 10.1038/ng.2982 PMC4064254

[acel12386-bib-0139] Shmulevich I , Dougherty ER , Kim S , Zhang W (2002) Probabilistic Boolean networks: a rule‐based uncertainty model for gene regulatory networks. Bioinformatics 18, 261–274. 11847074 10.1093/bioinformatics/18.2.261

[acel12386-bib-0140] Smith JD (2002) Apolipoproteins and aging: emerging mechanisms. Ageing Res. Rev. 1, 345–365. 12067591 10.1016/s1568-1637(02)00005-3

[acel12386-bib-0141] Sozou PD , Kirkwood TB (2001) A stochastic model of cell replicative senescence based on telomere shortening, oxidative stress, and somatic mutations in nuclear and mitochondrial DNA. J. Theor. Biol. 213, 573–586. 11742526 10.1006/jtbi.2001.2432

[acel12386-bib-0142] Steuer R (2006) Review: on the analysis and interpretation of correlations in metabolomic data. Brief. Bioinform. 7, 151–158. 16772265 10.1093/bib/bbl009

[acel12386-bib-0143] Subramanian A , Tamayo P , Mootha VK , Mukherjee S , Ebert BL , Gillette MA , Paulovich A , Pomeroy SL , Golub TR , Lander ES , Mesirov JP (2005) Gene set enrichment analysis: a knowledge‐based approach for interpreting genome‐wide expression profiles. Proc. Natl Acad. Sci. U. S. A. 102, 15545–15550. 16199517 10.1073/pnas.0506580102 PMC1239896

[acel12386-bib-0144] Suhre K , Meisinger C , Döring A , Altmaier E , Belcredi P , Gieger C , Chang D , Milburn MV , Gall WE , Weinberger KM , Mewes H‐W , Hrabé de Angelis M , Wichmann H‐E , Kronenberg F , Adamski J , Illig T (2010) Metabolic footprint of diabetes: a multiplatform metabolomics study in an epidemiological setting. PLoS ONE 5, e13953. 21085649 10.1371/journal.pone.0013953 PMC2978704

[acel12386-bib-0145] Swardfager W , Lanctôt K , Rothenburg L , Wong A , Cappell J , Herrmann N (2010) A meta‐analysis of cytokines in Alzheimer's disease. Biol. Psychiatry 68, 930–941. 20692646 10.1016/j.biopsych.2010.06.012

[acel12386-bib-0146] Tacutu R , Shore DE , Budovsky A , de Magalhães JP , Ruvkun G , Fraifeld VE , Curran SP (2012) Prediction of *C. elegans* longevity genes by human and worm longevity networks. PLoS ONE 7, 4–12. 10.1371/journal.pone.0048282 PMC3483217 23144747

[acel12386-bib-0147] Tacutu R , Craig T , Budovsky A , Wuttke D , Lehmann G , Taranukha D , Costa J , Fraifeld VE , de Magalhães JP (2013) Human ageing genomic resources: integrated databases and tools for the biology and genetics of ageing. Nucleic Acids Res. 41, D1027–D1033. 23193293 10.1093/nar/gks1155 PMC3531213

[acel12386-bib-0148] Teicher BA , Linehan WM , Helman LJ (2012) Targeting cancer metabolism. Clin. Cancer Res. 18, 5537–5545. 23071355 10.1158/1078-0432.CCR-12-2587 PMC3475613

[acel12386-bib-0149] The Gtex Consortium (2013) The genotype‐tissue expression (GTEx) project. Nat. Genet. 45, 580–585. 23715323 10.1038/ng.2653 PMC4010069

[acel12386-bib-0150] The Human Microbiome Project (2014a) NIH Human Microbiome Project defines normal bacterial makeup of the body. Available at: http://www.nih.gov/news/health/jun2012/nhgri-13.htm [Accessed June 3, 2014].

[acel12386-bib-0151] The Human Microbiome Project (2014b) Reference Genomes Data. Available at: http://www.hmpdacc.org/HMRGD.

[acel12386-bib-0152] Tur I , Castelo R (2012) Learning high‐dimensional mixed graphical models with missing values In Proceedings of the Sixth European Workshop on Probabilistic Graphical Models (A Cano, M Gomez‐Olmedo, TD Nielsen ed.). Granada: DECSAI, University of Granada, pp. 323–330.

[acel12386-bib-0153] Turnbaugh PJ , Ley RE , Hamady M , Fraser‐Liggett CM , Knight R , Gordon JI (2007) The human microbiome project. Nature 449, 804–810. 17943116 10.1038/nature06244 PMC3709439

[acel12386-bib-0154] Tzoulaki I , Ebbels TMD , Valdes A , Elliott P , Ioannidis JPA (2014) Design and analysis of metabolomics studies in epidemiological research: a primer on ‐omic technologies. Am. J. Epidemiol. 180, 129–139. Available at: http://www.ncbi.nlm.nih.gov/pubmed/24966222 [Accessed June 30, 2014]. 24966222 10.1093/aje/kwu143

[acel12386-bib-0155] Valdes AM , Glass D , Spector TD (2013) Omics technologies and the study of human ageing. Nat. Rev. Genet. 14, 601–607. 23938363 10.1038/nrg3553

[acel12386-bib-0156] Varki A , Cummings R , Esko J , Freeze H , Stanley P , Bertozzi C , Hart G , Etzler M (2009) Essentials of Glycobiology, 2nd edn The Consortium of Glycobiology Editors, ed., Cold Spring habour, NY: Cold Spring Harbor Laboratory; Auflage: 0002. Available at: http://www.ncbi.nlm.nih.gov/books/NBK1918/?report=reader [Accessed June 11, 2014]. 20301239

[acel12386-bib-0157] Vijg J , Suh Y (2005) Genetics of longevity and aging. Annu. Rev. Med. 56, 193–212. 15660509 10.1146/annurev.med.56.082103.104617

[acel12386-bib-0158] Wainwright MJ , Ravikumar P , Lafferty JD (2006) High‐dimensional graphical model selection using l1 ‐regularized logistic regression In Neural Information Processing Systems. (Schölkopf B. and Platt J.C. and Hoffman T., ed). Vancouver: MIT Press, pp 1465–1472.

[acel12386-bib-0159] Wang J , Zhang S , Wang Y , Chen L , Zhang X‐S (2009) Disease‐aging network reveals significant roles of aging genes in connecting genetic diseases. PLoS Comput. Biol. 5, e1000521. 19779549 10.1371/journal.pcbi.1000521 PMC2739292

[acel12386-bib-0160] Warming L , Hassager C , Christiansen C (2002) Changes in bone mineral density with age in men and women: a longitudinal study. Osteoporos. Int. 13, 105–112. 11905520 10.1007/s001980200001

[acel12386-bib-0161] Weidner CI , Lin Q , Koch CM , Eisele L , Beier F , Ziegler P , Bauerschlag DO , Jöckel K‐H , Erbel R , Mühleisen TW , Zenke M , Brümmendorf TH , Wagner W (2014) Aging of blood can be tracked by DNA methylation changes at just three CpG sites. Genome Biol. 15, R24. 24490752 10.1186/gb-2014-15-2-r24 PMC4053864

[acel12386-bib-0162] Weinert BT , Timiras PS (2003) Invited review: theories of aging. J. Appl. Physiol. 95, 1706–1716. 12970376 10.1152/japplphysiol.00288.2003

[acel12386-bib-0163] West J , Beck S , Wang X , Teschendorff AE (2013) An integrative network algorithm identifies age‐associated differential methylation interactome hotspots targeting stem‐cell differentiation pathways. Sci. Rep. 3, 1630. 23568264 10.1038/srep01630 PMC3620664

[acel12386-bib-0164] Wieser D , Papatheodorou I , Ziehm M , Thornton JM (2011) Computational biology for ageing. Philos. Trans. R. Soc. Lond. B Biol. Sci. 366, 51–63. 21115530 10.1098/rstb.2010.0286 PMC3001313

[acel12386-bib-0165] Wilhelm M , Schlegl J , Hahne H , Moghaddas Gholami A , Lieberenz M , Savitski MM , Ziegler E , Butzmann L , Gessulat S , Marx H , Mathieson T , Lemeer S , Schnatbaum K , Reimer U , Wenschuh H , Mollenhauer M , Slotta‐Huspenina J , Boese J‐H , Bantscheff M , Gerstmair A , Faerber F , Kuster B (2014) Mass‐spectrometry‐based draft of the human proteome. Nature 509, 582–587. 24870543 10.1038/nature13319

[acel12386-bib-0166] Willcox BJ , Donlon TA , He Q , Chen R , Grove JS , Yano K , Masaki KH , Willcox DC , Rodriguez B , Curb JD (2008) FOXO3A genotype is strongly associated with human longevity. Proc. Natl Acad. Sci. U. S. A. 105, 13987–13992. 18765803 10.1073/pnas.0801030105 PMC2544566

[acel12386-bib-0167] Wishart DS , Jewison T , Guo AC , Wilson M , Knox C , Liu Y , Djoumbou Y , Mandal R , Aziat F , Dong E , Bouatra S , Sinelnikov I , Arndt D , Xia J , Liu P , Yallou F , Bjorndahl T , Perez‐Pineiro R , Eisner R , Allen F , Neveu V , Greiner R , Scalbert A (2013) HMDB 3.0–the human metabolome database in 2013. Nucleic Acids Res. 41, D801–D807. 23161693 10.1093/nar/gks1065 PMC3531200

[acel12386-bib-0168] Xenarios I , Salwínski L , Duan XJ , Higney P , Kim S‐M , Eisenberg D (2002) DIP, the database of interacting proteins: a research tool for studying cellular networks of protein interactions. Nucleic Acids Res. 30, 303–305. 11752321 10.1093/nar/30.1.303 PMC99070

[acel12386-bib-0169] Xia J , Wishart DS (2010) MSEA: a web‐based tool to identify biologically meaningful patterns in quantitative metabolomic data. Nucleic Acids Res. 38, W71–W77. 20457745 10.1093/nar/gkq329 PMC2896187

[acel12386-bib-0170] Xia K , Xue H , Dong D , Zhu S , Wang J , Zhang Q , Hou L , Chen H , Tao R , Huang Z , Fu Z , Chen Y‐G , Han J‐DJ (2006) Identification of the proliferation/differentiation switch in the cellular network of multicellular organisms. PLoS Comput. Biol. 2, e145. 17166053 10.1371/journal.pcbi.0020145 PMC1664705

[acel12386-bib-0171] Xue H , Xian B , Dong D , Xia K , Zhu S , Zhang Z , Hou L , Zhang Q , Zhang Y , Han J‐DJ (2007) A modular network model of aging. Mol. Syst. Biol. 3, 147. 18059442 10.1038/msb4100189 PMC2174624

[acel12386-bib-0172] Yang J‐H , Li J‐H , Jiang S , Zhou H , Qu L‐H (2013) ChIPBase: a database for decoding the transcriptional regulation of long non‐coding RNA and microRNA genes from ChIP‐Seq data. Nucleic Acids Res. 41, D177–D187. 23161675 10.1093/nar/gks1060 PMC3531181

[acel12386-bib-0173] Yu H , Kim PM , Sprecher E , Trifonov V , Gerstein M (2007) The importance of bottlenecks in protein networks: correlation with gene essentiality and expression dynamics. PLoS Comput. Biol. 3, e59. 17447836 10.1371/journal.pcbi.0030059 PMC1853125

[acel12386-bib-0174] Yu Z , Zhai G , Singmann P , He Y , Xu T , Prehn C , Römisch‐Margl W , Lattka E , Gieger C , Soranzo N , Heinrich J , Standl M , Thiering E , Mittelstraß K , Wichmann H‐E , Peters A , Suhre K , Li Y , Adamski J , Spector TD , Illig T , Wang‐Sattler R (2012) Human serum metabolic profiles are age dependent. Aging Cell 11, 960–967. 22834969 10.1111/j.1474-9726.2012.00865.x PMC3533791

[acel12386-bib-0175] Yuan M , Lin Y (2007) Model selection and estimation in the Gaussian graphical model. Biometrika 94, 19–35.

[acel12386-bib-0176] Yuan Y , Li C‐T , Windram O (2011) Directed partial correlation: inferring large‐scale gene regulatory network through induced topology disruptions. PLoS ONE 6, e16835. 21494330 10.1371/journal.pone.0016835 PMC3071805

[acel12386-bib-0177] Zhang B , Horvath S (2005) A general framework for weighted gene co‐expression network analysis. Stat. Appl. Genet. Mol. Biol. 4, Article17. 10.2202/1544-6115.1128 16646834

[acel12386-bib-0178] Zhu B , Wang X , Li L (2010) Human gut microbiome: the second genome of human body. Protein Cell 1, 718–725. 21203913 10.1007/s13238-010-0093-z PMC4875195

[acel12386-bib-0179] Ziller MJ , Gu H , Müller F , Donaghey J , Tsai LT‐Y , Kohlbacher O , De Jager PL , Rosen ED , Bennett DA , Bernstein BE , Gnirke A , Meissner A (2013) Charting a dynamic DNA methylation landscape of the human genome. Nature 500, 477–481. 23925113 10.1038/nature12433 PMC3821869

